# The Relationship Between Postoperative Pain and Subcutaneous Tissue Thickness Following Cesarean Section

**DOI:** 10.3390/jcm15145731

**Published:** 2026-07-22

**Authors:** Mustafa Bakırcı, Çağlayan Ateş, Hüseyin Karakaya, Ece Ermin

**Affiliations:** Department of Obstetrics and Gynecology, Faculty of Medicine, Yozgat Bozok University, 66100 Yozgat, Türkiye; caglayan.ates@bozok.edu.tr (Ç.A.); huseyin.karakaya@bozok.edu.tr (H.K.); ece.ermin@bozok.edu.tr (E.E.)

**Keywords:** cesarean section, postoperative pain, subcutaneous tissue thickness, body mass index, acute pain, good health and well-being

## Abstract

**Objective:** The aim of this study is to identify clinical and obstetric factors associated with acute postoperative pain in pregnant women undergoing primary elective cesarean section and, in particular, to investigate the relationship between intraoperative subcutaneous tissue thickness and the severity of postoperative pain. **Materials and Methods:** This prospective cohort study included 82 pregnant women who underwent primary elective cesarean section between August 2025 and April 2026. All patients underwent cesarean delivery under spinal anesthesia by the same surgeon using a Pfannenstiel incision and received the clinic’s standard postoperative analgesia protocol. Subcutaneous tissue thickness was measured along the Pfannenstiel incision line during cesarean section using a sterile millimeter ruler. Postoperative pain intensity was assessed using the Visual Analog Scale (VAS) at 0, 2, 6, 12, and 24 h. Relationships between variables were examined using Spearman’s correlation analysis. Multivariate linear regression analyses were performed for the 12th- and 24th-hour VAS scores to evaluate the independent predictors of postoperative pain. **Results:** The participants’ mean age was 26.1 ± 4.6 years, and their mean BMI was 30.4 ± 5.1 kg/m^2^. The median subcutaneous tissue thickness was 20 mm (interquartile range (IQR): 15–25). No significant association was found between subcutaneous tissue thickness and postoperative pain scores or changes in hemoglobin levels (all *p* > 0.05). A moderate positive correlation was observed between subcutaneous tissue thickness and BMI (r = 0.501, *p* < 0.001) and fetal weight (r = 0.428, *p* < 0.001). In multivariate regression analyses, subcutaneous tissue thickness, age, BMI, fetal weight, gestational age, hemoglobin change, and parity were found not to be independent predictors of VAS scores at 12 or 24 h (all *p* > 0.05). **Conclusions:** Subcutaneous tissue thickness was not found to be associated with the severity of postoperative pain in patients undergoing primary elective cesarean section. Furthermore, none of the clinical and obstetric variables evaluated were shown to be independent predictors of acute postoperative pain. These findings suggest that local anatomical measurements alone may not be sufficient to explain postoperative pain following cesarean delivery and that postoperative pain is likely influenced by multiple non-anatomical factors.

## 1. Introduction

Despite guidelines and policies aimed at reducing cesarean section rates in many parts of the world, the cesarean delivery rate continues to rise [1]. As a result, cesarean section ranks among the most commonly performed surgical procedures [2,3]. Acute postoperative pain following cesarean delivery arises as a result of tissue damage associated with the surgery and uterine contractions [4]. Surgical procedures often lead to postoperative pain, and the effective and early management of this pain is of great importance for ensuring patient comfort, supporting the healing and rehabilitation process, and preventing potential complications [5]. Inadequate pain control following a cesarean section can lead to numerous adverse clinical outcomes, including delayed early mobilization, negative effects on breastfeeding, disruption of the mother-infant bond, prolonged hospital stay, and an increased risk of thromboembolic complications [6,7,8]. Additionally, inadequate control of acute postoperative pain is considered a significant risk factor for the development of chronic postoperative pain [9].

Post-cesarean pain is influenced by many factors [10]. Postoperative pain is a multifactorial process influenced by numerous variables, including maternal, fetal, and surgical factors, as well as anesthesia management [6]. In addition to maternal characteristics such as pain threshold, body mass index (BMI), age, educational level, and psychosocial status, obstetric conditions that cause uterine distension—such as fetal weight, gestational age, polyhydramnios, multiple pregnancy, and fetal macrosomia—can also affect pain intensity. Additionally, a history of previous childbirth and surgery, the presence of adhesions, surgical technique, duration of the procedure, type of incision, amount of intraoperative blood loss, choice of anesthesia, postoperative mobilization, hematoma, development of infection, and the breastfeeding process may also be determinants of postoperative pain [4].

Although BMI is the most commonly used parameter in the assessment of obesity, it is a systemic measurement and may not always adequately reflect the local characteristics of the surgical site. In contrast, abdominal subcutaneous tissue thickness is a parameter that more directly represents the local adipose tissue in the surgical incision area. Subcutaneous adipose tissue is not merely a passive energy storage tissue but also a metabolically active endocrine organ that secretes a variety of bioactive mediators. Increased adipose tissue is associated with a chronic low-grade inflammatory state characterized by increased secretion of pro-inflammatory adipokines, such as leptin, and cytokines including interleukin-6 (IL-6) and tumor necrosis factor-α (TNF-α), together with reduced secretion of the anti-inflammatory adipokine adiponectin. These mediators may contribute to postoperative pain by promoting peripheral inflammation, nociceptor sensitization, and alterations in wound healing [11,12]. In addition to these biological mechanisms, increased subcutaneous tissue thickness may complicate surgical dissection, increase tissue trauma, and consequently influence postoperative pain [3,4,13]. Thus, subcutaneous tissue thickness may affect postoperative pain through both biological and mechanical mechanisms.

While maternal, obstetric, and perioperative factors influencing post-cesarean pain have been extensively studied in the literature, the potential effect of subcutaneous tissue thickness at the surgical incision site on postoperative pain has not been sufficiently investigated [4,14]. Although several studies have investigated the association between obesity or BMI and postoperative pain, studies evaluating directly measured intraoperative subcutaneous tissue thickness in relation to postoperative pain following cesarean delivery remain limited [15]. Furthermore, BMI is an indirect anthropometric measure that does not reflect the regional distribution of adipose tissue or the local tissue characteristics at the surgical incision site. Therefore, direct intraoperative assessment of subcutaneous tissue thickness at the cesarean incision site may provide clinically relevant information beyond BMI and improve our understanding of factors contributing to postoperative pain.

Subcutaneous tissue thickness can be assessed using imaging modalities such as ultrasonography or computed tomography [16]. However, these methods may be influenced by imaging protocols, operator experience, and the anatomical site selected for measurement [17]. In contrast, intraoperative direct measurement allows assessment of the actual subcutaneous tissue thickness at the surgical incision site without additional imaging. Accordingly, subcutaneous tissue thickness was measured directly during surgery in the present study.

Despite growing evidence on the relationship between obesity and postoperative pain, the potential contribution of directly measured subcutaneous tissue thickness at the cesarean incision site remains unclear. Addressing this knowledge gap may improve our understanding of whether local tissue characteristics provide additional information beyond conventional anthropometric measures when evaluating postoperative pain following cesarean delivery. The primary objective of this study is to identify the clinical and obstetric factors associated with acute postoperative pain in pregnant women undergoing primary elective cesarean section and, in particular, to investigate the relationship between intraoperative subcutaneous tissue thickness and the severity of postoperative pain. We hypothesized that greater intraoperative subcutaneous tissue thickness would be associated with greater postoperative pain severity following primary elective cesarean delivery.

## 2. Materials and Methods

This prospective cohort study was conducted at a tertiary university hospital between August 2025 and April 2026. The study was initiated after approval was granted by the local ethics committee of Yozgat Bozok University (No: 2025-GOKAEK-2513_2025.07.02_510, date: 2 July 2025). Written and verbal informed consent was obtained from all participants before study enrollment. All authors have acted in accordance with the Declaration of Helsinki.

### 2.1. Participants

Pregnant women aged 18–45 who were scheduled for a primary elective cesarean section were included in the study. Patients with a history of pelvic or abdominal surgery, multiple pregnancies, polyhydramnios, abnormal fetal presentation, premature rupture of membranes, systemic diseases such as diabetes mellitus or hypertension, a history of psychiatric disorders (including depression or anxiety), smokers, those with a history of chronic pain, or those who developed intraoperative complications were excluded from the study.

### 2.2. Surgical Procedure

To minimize surgical variability, all operations were performed by a single surgeon under spinal anesthesia using a standard technique; cases with an operative time exceeding 60 min were excluded from the analysis. Prior to the skin incision, the abdominal area was disinfected with a 10% povidone-iodine solution in accordance with standard protocols. A Pfannenstiel incision was performed in all cases. After incising the skin and subcutaneous tissues with a scalpel, the fascia was opened, and access to the abdominal cavity was gained. The bladder peritoneum was dissected to reach the uterus from the lower uterine segment, and the fetus was delivered. Following the removal of the placenta and its appendages, the uterine cavity was cleaned, and the uterus was closed with a single-layer continuous 1-0 Vicryl suture. The fascia was closed with a continuous 1-0 Vicryl suture, and the skin was closed subcutaneously with 3-0 Monocryl.

### 2.3. Data Collection

Patients were admitted to the ward on the day of their scheduled cesarean section, and demographic and clinical data were recorded on a standard data collection form. Information regarding age, obstetric history, and weight gain during pregnancy was recorded. Body weight (kg) and height (m) were measured, and BMI was calculated in kg/m^2^.

Waist circumference measurements were taken using a standard tape measure at both the umbilical level and the incision level, with the patient standing in the standard position at the end of expiration.

Subcutaneous tissue thickness was determined by measuring the distance from the skin surface to the fascia along the Pfannenstiel incision line during cesarean section, using a sterile millimeter-marked metal ruler held perpendicular to the fascia, with measurements taken in millimeters. All measurements were performed by the same surgeon using the same method and measurement tool. Additionally, incision length and newborn birth weight (g) were recorded. Patients’ hemoglobin levels were measured and recorded on the day of hospital admission (preoperative) and at 2, 6, and 24 h postoperatively (g/dL). The change in hemoglobin was calculated as the difference between the preoperative hemoglobin value and the corresponding postoperative measurement.

### 2.4. Pain Assessment and Analgesia Protocol

Postoperative pain intensity was assessed using the Visual Analog Scale (VAS). The VAS consists of a 100 mm horizontal line, with one end representing “no pain” and the other end representing “most severe pain.” Patients indicate their pain level by marking a point on this line, and the distance from the mark to the “no pain” point is measured in millimeters. Higher scores indicate increased pain intensity [18].

Postoperative pain intensity was assessed at rest using the VAS at 0, 2, 6, 12, and 24 h postoperatively. Pain was self-reported by the patient, and the VAS score was recorded by the ward nurse. The nurses performing the pain assessments were blinded to the intraoperative subcutaneous tissue thickness measurements. The primary endpoint was the 24 h VAS score, and the secondary endpoint was the 12 h VAS score.

A standard postoperative analgesia protocol was applied to all patients. All VAS assessments were performed before analgesic administration at the corresponding postoperative time point. Intravenous dexketoprofen (50 mg) was administered after the VAS assessments at postoperative 0 and 12 h. Patients with a VAS score ≥5 at postoperative 6 h received intravenous paracetamol (1 g) as rescue analgesia. The need for rescue analgesia was recorded.

### 2.5. Statistical Analysis

The sample size was calculated using G*Power software (version 3.1.9.6; Heinrich Heine University, Düsseldorf, Germany) based on an expected correlation coefficient of r = 0.30, a significance level of α = 0.05, and 80% power (1 − β), and was determined to be a minimum of 82 patients. The sample size calculation was based on the primary correlation analysis, which was the primary objective of the study.

The distribution of continuous variables was assessed using the Shapiro–Wilk test. Variables were expressed as mean ± standard deviation or median (interquartile range), depending on their distribution. Relationships between variables were analyzed using the Spearman rank correlation coefficient. Multivariate linear regression analyses were performed to evaluate the independent predictors of postoperative pain. In these analyses, the 12 h and 24 h VAS scores were used as the dependent variables. In both models, subcutaneous tissue thickness, age, BMI, fetal weight, gestational age, and parity were included as independent variables. Additionally, the change in hemoglobin levels at 24 h postoperatively was included in the model created for the 24 h VAS score. Since hemoglobin measurements were not available at 12 h postoperatively, the hemoglobin change was evaluated only for the 24 h model. Model variables were determined by considering their clinical significance and factors reported in the literature to be associated with postoperative pain. A *p*-value of <0.05 was considered statistically significant.

## 3. Results

The study was completed with a total of 82 patients who met the inclusion criteria and were included in the analysis. The mean age of the participants was 26.1 ± 4.6 years, and the mean BMI was 30.4 ± 5.1 kg/m^2^. The median subcutaneous tissue thickness was 20 mm (IQR: 15–25). The median VAS score at baseline was 3 (IQR: 2–4), while the median VAS score at 24 h postoperatively was 0 (IQR: 0–2) (Table 1). Four patients (4.9%) required rescue analgesia with intravenous paracetamol after reaching a VAS score of ≥5 at postoperative 6 h.

**Figure 1:**
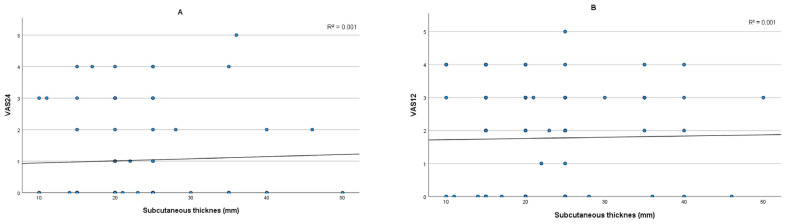
Relationship between subcutaneous tissue thickness and postoperative pain intensity. (**A**) VAS at 24 h; (**B**) VAS at 12 h. No clear association was observed at either time point (R^2^ = 0.001 and R^2^ = 0.0004, respectively).

The results of the Spearman correlation analysis revealed no significant association between subcutaneous tissue thickness and changes in postoperative VAS scores or hemoglobin levels (all *p* > 0.05). However, a moderate positive correlation was observed between subcutaneous tissue thickness and body mass index (r = 0.501, *p* < 0.001) and fetal weight (r = 0.428, *p* < 0.001) (Table 2 and Table 3) (Figure 1).

No consistent relationship was found between changes in hemoglobin levels and pain scores. Only a weak, negative correlation was observed between the baseline VAS score and changes in hemoglobin levels (VAS_0_ vs. hemoglobin change at 2 h: r = −0.225, *p* = 0.042; VAS_0_ vs. hemoglobin change at 24 h: r = −0.272, *p* = 0.013); all other correlations were not statistically significant (*p* > 0.05) (Table 3).

In the multivariate linear regression analysis conducted for pain intensity at 24 h postoperatively, the model was generally not statistically significant (F = 0.335, *p* = 0.936) and explained only a very small portion of the variance (R^2^ = 0.031, adjusted R^2^ = −0.061). No independent association between pain intensity and any of the evaluated variables, including subcutaneous tissue thickness, was detected (B = 0.021, 95% CI: −0.030–0.071, *p* = 0.414). Similarly, age, BMI, fetal weight, gestational age, hemoglobin levels, and parity were not significant predictors (all *p* > 0.05) (Table 4).

Similar findings were observed for early postoperative pain (at 12 h). The regression model was not statistically significant (F = 0.279, *p* = 0.945) and had very low explanatory power (R^2^ = 0.022, adjusted R^2^ = −0.056). No significant association was found between subcutaneous tissue thickness and VAS scores (B = −0.008, 95% CI: −0.061–0.045, *p* = 0.757), and no other variables showed an independent association (Table 5).

## 4. Discussion

In our study, factors associated with postoperative pain in patients undergoing primary elective cesarean section were evaluated, and the potential effect of subcutaneous tissue thickness on pain intensity was specifically investigated. The main finding was that subcutaneous tissue thickness was not associated with postoperative pain at either 12 or 24 h. Furthermore, it was determined that none of the clinical and obstetric variables evaluated—such as age, BMI, fetal weight, gestational age, hemoglobin levels, and parity—were independent predictors of pain intensity. These findings are consistent with both correlation analyses and multivariate regression results, suggesting that the structural parameters examined play a limited role in explaining postoperative pain.

Theoretically, it can be anticipated that increased subcutaneous tissue thickness may be associated with more extensive tissue dissection, increased surgical manipulation, and a more pronounced local inflammatory response [19]. It is thought that this could be a potential risk factor for postoperative pain, particularly in obese patients, as the surgical field may be technically more challenging and require more extensive tissue manipulation [20]. However, there are no consistent findings in the literature regarding this issue. While it has been reported that abdominal obesity is associated with early-stage pain scores and analgesic requirements in cases of laparoscopic cholecystectomy [19], it has been shown that high BMI values are not associated with the severity of postoperative pain in patients undergoing elective hernia surgery [21]. In a recent cohort study analyzing data from 808 patients who underwent various surgical procedures, contrary to expectations, no clinically significant association was found between high BMI values and postoperative pain scores or cumulative opioid consumption [22]. The current literature suggests that the effect of BMI and obesity on postoperative pain is inconsistent and that this relationship may vary depending on the procedure and patient characteristics. In our study as well, the finding that neither BMI nor subcutaneous tissue thickness was associated with postoperative pain supports this view. The absence of an observed association in our cohort may reflect the multifactorial nature of postoperative pain. Although greater subcutaneous tissue thickness may theoretically increase tissue trauma and local inflammatory responses, the relationship between subcutaneous tissue thickness and postoperative pain may be influenced by a complex interplay of anatomical and non-anatomical factors, including psychological factors and individual pain perception. In addition, the relatively homogeneous study population and the use of a standardized surgical technique and a uniform postoperative analgesia protocol may have reduced between-patient variability, making a modest association more difficult to detect.

In a study conducted in China that evaluated various types of surgeries, including cesarean sections, female gender, a history of chronic pain prior to surgery, daily opioid use before hospitalization, longer surgery duration, and a high BMI were identified as significant factors associated with acute postoperative pain [23]. It is emphasized that clinical management and patient characteristics may be more decisive in the perception of acute pain after cesarean section than anatomical measurements. In a prospective study involving 290 cesarean section patients, the majority of whom were emergency cases, it was reported that the independent factors predicting moderate-to-severe pain within the first 24 h were preoperative anxiety, history of prior surgery, Pfannenstiel incision type, and the absence of regional anesthesia; whereas systemic anthropometric measurements were not found to be associated with pain [24]. Similarly, other studies have shown that operative duration, type of anesthesia, analgesic agents used, preoperative anxiety, and history of prior surgery are associated with postoperative pain [25,26]. A recent review also identifies a history of chronic pain, inadequate regional analgesia, and preoperative anxiety as significant risk factors for moderate-to-severe postoperative pain [8]. When these findings are considered together, they suggest that acute pain following cesarean section cannot be explained solely by anatomical or anthropometric characteristics. In our study as well, the lack of association between subcutaneous tissue thickness and BMI with postoperative pain suggests that unassessed clinical, psychological, and perioperative factors may also play a role in the onset of acute postoperative pain. These factors may have a greater influence on postoperative pain than local anatomical characteristics and should be considered in future studies investigating predictors of pain following cesarean delivery.

In our study, no clinically significant or consistent association was found between changes in hemoglobin levels and postoperative pain. A randomized controlled trial conducted in cesarean section patients reported that the use of an abdominal binder both reduced pain scores and helped maintain higher postoperative hemoglobin and hematocrit levels. However, the direct relationship between hemoglobin levels and pain was not evaluated in that study [27]. In our study, only weak negative correlations were found between baseline pain scores and changes in hemoglobin levels; however, these relationships did not persist in multivariate analyses. These findings suggest that postoperative hemoglobin changes are not an independent predictor of acute postoperative pain. Similarly, the fact that obstetric variables such as fetal weight, gestational age, and parity were not identified as independent predictors indicates that the clinical and obstetric variables evaluated may have a limited contribution to explaining the severity of postoperative pain.

Given that the literature reports quite high prevalence rates of moderate to severe pain in the first 24 h following cesarean section, ranging from 20% to 89.8% [8,23,24,25,26], understanding the factors influencing this pain may play a significant role in pain reduction. The findings of our study suggest that structural parameters such as local subcutaneous tissue thickness and BMI are not primary determinants of postoperative pain and indicate that future studies focusing on psychological, neurophysiological, and perioperative factors may be more beneficial.

One of the study’s key strengths is its prospective design. Additionally, maintaining a homogeneous patient group, including only primary elective cesarean sections, having all surgeries performed by the same surgeon, and applying a standard analgesia protocol are significant methodological strengths. Furthermore, measuring subcutaneous tissue thickness directly during surgery allowed for an objective assessment of local anatomical features. The fact that pain was assessed at multiple time points further enhances the study’s methodological strength.

However, the study has some limitations. First, the study was conducted at a single center, and the sample size is relatively small. Additionally, the inclusion of only cases undergoing primary elective cesarean sections may limit the generalizability of the results to emergency cesarean sections or more heterogeneous obstetric populations. In addition, the sample size was calculated to detect a moderate correlation (r = 0.30). Therefore, weaker but potentially clinically meaningful associations, particularly those evaluated in the multivariable regression analyses, may not have been detected. Second, psychological factors such as anxiety, pain catastrophizing, preoperative expectations, depressive symptoms, and individual pain sensitivity were not assessed. These factors have been consistently identified as important predictors of postoperative pain and may have influenced the pain scores observed in our study. In addition, other patient-related factors, such as socioeconomic status, sleep quality, and breastfeeding at the time of pain assessment, were not evaluated. Third, the need for additional postoperative analgesics or total analgesic consumption was not evaluated. Additionally, only the first 24 h acute postoperative period was examined, and the development of chronic postoperative pain was not investigated. Furthermore, incision pain and pain related to uterine contractions were not evaluated separately; therefore, the VAS scores reflected the patients’ overall postoperative pain. Finally, the relatively homogeneous BMI distribution of our cohort may have limited our ability to detect an association between subcutaneous tissue thickness and postoperative pain. Future studies including women with a wider range of BMI values and subcutaneous tissue thickness are needed to determine whether different findings are observed in more severely obese populations.

## 5. Conclusions

In conclusion, this study demonstrated that subcutaneous tissue thickness is not associated with the severity of postoperative pain following cesarean section. Furthermore, it was found that the clinical and obstetric variables evaluated—such as age, BMI, fetal weight, gestational age, changes in hemoglobin levels, and parity—were not independent predictors of postoperative pain. These findings suggest that local anatomical measurements alone may not be sufficient to explain acute postoperative pain. Future studies with larger sample sizes that evaluate psychological, clinical, and biological factors together may contribute to a more comprehensive understanding of the determinants of post-cesarean pain.

## Figures and Tables

**Table 1:** Demographic and clinical characteristics of the study population.

Variable	Value
Age (years)	26.1 ± 4.6
BMI (kg/m^2^)	30.4 ± 5.1
Gestational week	39.1 ± 1.1
Gestational weight gain (kg)	13.6 ± 6.8
Fetal weight (g)	3214 ± 480
Waist circumference (umbilical) (cm)	108.3 ± 14.0
Waist circumference (incision level) (cm)	107.2 ± 14.2
Incision length (mm)	138 ± 12
Subcutaneous thickness (mm)	20 (15–25)
Hemoglobin change (2 h)	0.92 ± 0.71
Hemoglobin change (6 h)	1.51 ± 0.82
Hemoglobin change (24 h)	2.13 ± 0.91
Parity (multipar), n (%)	14 (17.1%)
VAS_0_	3 (2–4)
VAS_2_	2 (0–3)
VAS_6_	2 (0–3)
VAS_12_	2 (0–3)
VAS_24_	0 (0–2)

VAS: Visual Analog Scale; BMI: Body Mass Index.

**Table 2:** Correlation between clinical variables and pain (VAS) scores.

	Spearman Rho	VAS_0_	VAS_2_	VAS_6_	VAS_12_	VAS_24_
Subcutaneous thickness	Rho	0.076	0.035	0.058	−0.027	0.079
	*p* value	0.495	0.755	0.607	0.807	0.482
BMI	Rho	0.006	−0.038	−0.049	0.050	−0.039
	*p* value	0.954	0.734	0.660	0.656	0.726
Fetal weight	Rho	−0.113	−0.015	−0.168	0.091	−0.029
	*p* value	0.311	0.892	0.132	0.418	0.799
Gestational weight gain	Rho	−0.107	−0.072	−0.011	0.126	−0.051
	*p* value	0.339	0.521	0.922	0.260	0.650
Gestational week	Rho	−0.029	0.007	−0.168	−0.040	0.017
	*p* value	0.797	0.951	0.131	0.721	0.881

VAS: Visual Analog Scale; BMI: Body Mass Index.

**Table 3:** Correlation between pain scores and hemoglobin changes.

	Spearman Rho	VAS_0_	VAS_2_	VAS_6_	VAS_12_	VAS_24_
Hb change (2 h)	Rho	−0.225	−0.045	−0.097	0.050	−0.071
	*p* value	0.042	0.687	0.386	0.653	0.528
Hb change (6 h)	Rho	−0.190	−0.045	0.023	−0.017	0.020
	*p* value	0.087	0.691	0.838	0.879	0.855
Hb change (24 h)	Rho	−0.272	0.018	−0.136	0.106	−0.093
	*p* value	0.013	0.873	0.223	0.343	0.407

VAS: Visual Analog Scale; Hb: Hemoglobin.

**Table 4:** Multivariable linear regression analysis for postoperative pain intensity (VAS at 24 h).

Variable	B	95% CI	β	*p* Value
Age	0.002	−0.078 to 0.081	0.005	0.969
Gestational week	0.083	−0.291 to 0.457	0.063	0.659
Subcutaneous thickness (mm)	0.021	−0.030 to 0.071	0.118	0.414
Fetal weight	−0.001	−0.002 to 0.001	−0.166	0.336
Hemoglobin change (24 h)	0.045	−0.337 to 0.428	0.028	0.814
BMI	−0.009	−0.095 to 0.077	−0.031	0.839
Parity (multipar vs. nullipar)	0.432	−0.506 to 1.370	0.112	0.362

B: Unstandardized regression coefficient; CI: confidence interval; BMI: body mass index. Model statistics: R^2^ = 0.031, Adjusted R^2^ = −0.061, F = 0.335, *p* = 0.936.

**Table 5:** Multivariable linear regression analysis for postoperative pain intensity (VAS at 12 h).

Variable	B	95% CI	β	*p* Value
Age	0.011	−0.072–0.095	0.034	0.786
Gestational week	−0.166	−0.553–0.221	−0.118	0.396
Subcutaneous thickness	−0.008	−0.061–0.045	−0.045	0.757
Fetal weight	0.001	−0.001–0.002	0.174	0.298
BMI	−0.005	−0.096–0.085	−0.018	0.905
Parity (multipar vs. nullipar)	−0.317	−1.303–0.670	−0.077	0.524

B: Unstandardized regression coefficient; CI: confidence interval; BMI: body mass index. Model statistics: R^2^ = 0.022, Adjusted R^2^ = −0.056, F = 0.279, *p* = 0.945.

## Data Availability

Data are available upon reasonable request from the corresponding author.

## Abbreviations

The following abbreviations are used in this manuscript:

BMI	Body Mass Index
CI	Confidence Interval
IQR	Interquartile Range
VAS	Visual Analog Scale
